# Transcriptional regulatory factor *AHA_4052* regulates aminoglycoside resistance in *Aeromonas hydrophila*

**DOI:** 10.3389/fmicb.2025.1689335

**Published:** 2025-11-25

**Authors:** Wanxin Li, ZhiWei Tu, Huaiyao Zhang, Binrong Lu, Fang Liu, Xiangmin Lin, Guibin Wang

**Affiliations:** 1 Institute of Population Medicine, School of Public Health, Fujian Medical University, Fuzhou, China; 2 State Key Laboratory of Medical Proteomics, Beijing Proteome Research Center, National Center for Protein Sciences (Beijing), Beijing Institute of Lifeomics, Beijing, China; 3 Fujian Provincial Key Laboratory of Agroecological Processing and Safety Monitoring, College of Life Sciences, Fujian Agriculture and Forestry University, Fuzhou, China; 4 Key Laboratory of Marine Biotechnology of Fujian Province, Institute of Oceanology, Fujian Agriculture and Forestry University, Fuzhou, China; 5 Changting Branch of Longyan Tobacco Company, Longyan, Fujian, China

**Keywords:** *Aeromonas hydrophila*, OmpR/PhoB family, aminoglycosides, antibiotic resistance, ChIP-PCR

## Abstract

**Introduction:**

With the increasing identification of multidrug resistance in Aeromonas hydrophila isolated from diverse food sources, there is an urgent need to investigate its resistance mechanism. Previous studies have demonstrated that OmpR/PhoB-type response regulators (RRs) play critical roles in mediating bacterial tolerance to various environmental stresses.

**Methods and results:**

In this study, we constructed an *AHA_4052* gene knockout strain, a member of the OmpR/PhoB family of DNA-binding response regulators, and studied its phenotypic characteristics. Deletion of the *AHA_4052* gene rendered the bacteria significantly more sensitive to high temperature, osmotic stress, and aminoglycoside antibiotics compared to the wild-type *A. hydrophila*. Label-free quantitative proteomic analysis revealed 131 differentially expressed proteins in the *ΔAHA_4052* mutant strain. These proteins were predominantly associated with ribosome, butanoate metabolism, and glycerophospholipid metabolism pathways, with ribosome-related proteins accounting for 17.56% of the total. Additionally, seven antibiotic resistance-related proteins exhibited significant expression changes in the mutant strain. Chromatin immunoprecipitation assay–polymerase chain reaction (ChIP-PCR) validation further demonstrated that the transcriptional regulator *AHA_4052* directly binds to the promoters of two resistance genes, AHA_2114 and AHA_3488.

**Discussion:**

Collectively, these findings indicate that the transcriptional regulatory factor *AHA_4052* plays a critical role in stress tolerance, particularly against aminoglycosides, providing insights into the resistance mechanisms of *A. hydrophila* and potentially informing the development of new therapeutic strategies.

## Introduction

With the continuous increase in global fish consumption, rising from 9.0 kg per capita in 1961 to 20.5 kg in 2018, the food safety of aquatic products has attracted increasing attention (Ibrahim et al., 2020). *Aeromonas hydrophila*, a Gram-negative pathogen widely distributed in aquatic environments, not only directly causes septicemia in fish but also serves as an emerging opportunistic human pathogen responsible for gastroenteritis, wound infections, and even life-threatening septicemia in immunocompromised individuals (Pessoa et al., 2022; Wang T. et al., 2022). The incidence of *A. hydrophila* infections peaks during summer, and its incidence has remained high in recent years. For instance, infection rates have been reported to reach 47% in farmed fish in Egypt and exceed 30% in retail fish from the United States, India, and China, highlighting its global distribution and significance as a foodborne hazard (Dahdouh et al., 2016; Yang et al., 2018; Jeamsripong et al., 2025).

In the field of aquaculture, the extensive use of antibiotics to control *A. hydrophila* has driven increased antibiotic resistance and the emergence of multidrug-resistant strains. The situation is further exacerbated by the fact that *A. hydrophila* possesses the capability to form biofilms (De Silva and Heo, 2022), which significantly enhances its resilience and persistence in the environment, making its prevention and control problem even more complex and difficult to solve. Previous studies have reported that *A. hydrophila* was resistant to ampicillin, tetracycline, streptomycin, nalidixic acid, and kanamycin, and multiple studies have reported that the resistance rate of *A. hydrophila* isolates to ampicillin reached 100% (Yang et al., 2018; Borella et al., 2020; Kaur et al., 2024). However, the potential molecular mechanisms underlying its resistance, particularly the complex interactions between intrinsic resistance genes, metabolic pathways, regulatory networks, and signal transduction-mediated tolerance, have not yet been fully elucidated. Therefore, there is an urgent need not only to elucidate the antibiotic resistance mechanisms of *A. hydrophila* but also to provide information for developing new and targeted strategies to control the pathogen.

Two-component systems (TCSs) composed of a histidine kinase (HK) and response regulator (RR) are ubiquitous in bacteria. They are essential for maintaining cellular homeostasis and represent a crucial mechanism for many organisms to respond to environmental stress. For example, the EnvZ-OmpR TCS mediates osmotic stress adaptation by regulating porin expression (Cai and Inouye, 2002); the QseB/QseC system acts as a global regulator of quorum sensing (Zhu et al., 2023); and the PhoP-PhoQ TCS is involved in the regulation of bacterial Mg^2+^ homeostasis, resistance to pH and antimicrobial peptides, and mediates a response to heat, high osmotic pressure, oxide salt or bile salt, and toxicity (Groisman et al., 2021; Cabezudo et al., 2022; Mao et al., 2025). There are also many studies reporting that TCSs are associated with bacterial resistance and directly modulate resistance mechanisms, including cell surface modifications, changes in cell permeability, biofilm formation, and the expression of antibiotic-degrading enzymes (Tierney and Rather, 2019; Li et al., 2023; Long et al., 2025). Notable examples include the Vancomycin resistance-associated Sensor-Regulator (VraSR)-TCS, which regulates genes responsible for vancomycin resistance (Chen et al., 2016), the Polymyxin resistance-associated Sensor-Regulator (PmrAB)-TCS, which confers colistin resistance via QseBC modulation (Fernandez-Ciruelos et al., 2023), and the Biofilm formation-associated Sensor-Regulator (BfmRS)-TCS, which enhances bacterial resistance through biofilm formation (Cao et al., 2020). As TCSs have emerged as a potential target for antibacterial drug design, more than 40 TCSs related to bacterial drug resistance have been found (Tierney and Rather, 2019; De Gaetano et al., 2023).

Response regulators (RRs) are a major family of signaling proteins in prokaryotes that link diverse cellular activities in bacteria to environmental factors (Stock et al., 2000; Zhang et al., 2024). Among these, DNA-binding domains are the main types of RRs, accounting for approximately 60% of RRs, while OmpR/PhoB is the most dominant subfamily of DNA-binding domains, followed by NarL/FixJ and NtrC/DctD (Gao et al., 2007), highlighting their central role in transcriptional regulation. In *A. hydrophila*, the *AHA_4052* gene encodes a two-component system of regulatory proteins belonging to the OmpR/PhoB family of DNA-binding domains; however, its biological function remains clear. In this study, we generated an *AHA_4052* knockout strain via homologous recombination and studied its phenotypic characteristics. The mutant strain exhibited increased sensitivity to high temperature, osmotic stress, and aminoglycoside antibiotics. This prompted a comprehensive investigation into the underlying molecular mechanisms. To globally profile the regulatory network governed by *AHA_4052*, we used a label-free quantitative proteomic method to compare the differential expression of proteins between the *ΔAHA_4052* mutant and wild-type *A. hydrophila* strains. The results of mass spectrometry (MS) identification showed that a total of 131 differentially expressed proteins were detected, and the deletion of the *AHA_4052* gene resulted in significant changes in the ribosome, butanoate metabolism, and glycerophospholipid metabolism pathways. Notably, seven known or putative drug resistance-related proteins exhibited significant expression changes. ChIP-PCR validation confirmed direct binding of *AHA_4052* to the promoters of two candidate resistance genes, *AHA_3488* and *AHA_2114*. In conclusion, these findings advance our understanding of the *AHA_4052* gene’s role in aminoglycoside resistance and highlight its potential as a therapeutic target for combating drug-resistant *A. hydrophila*.

## Materials and methods

### Bacterial strains and culture conditions

The bacterial strains and plasmids used in this study are listed in Table 1. All strains were cultured in Luria–Bertani (LB) medium. Antibiotics were added to the culture medium at varying concentrations according to experimental requirements. *Aeromonas hydrophila* wild-type and gene knockout strains were cultured at 30 °C, while *Escherichia coli* MC1061 and S17-1 were grown at 37 °C.

**Table 1:** Bacterial strains and plasmids used in this study.

Strain or plasmid	Description	Source
Strains		
*Aeromonas hydrophila* ATCC7966	Wild-type	In our laboratory
*ΔAHA_4052*	*AHA_4052* deletion mutant from *A. hydrophila*	In this study
*ΔAHA_4052* + *AHA_4052*	*∆AHA_4052* complemented with pBBR-AHA_4052	In this study
*ΔAHA_4052* + vector	*∆AHA_4052* complemented with pBBR	In this study
MC1061	*E. coli* K-12, Str^R^, λpir	In our laboratory
S17-1	*E. coli* K-12, λpir.	In our laboratory
Plasmid		
pRE112	Suicide vector, Cm^r^, *sacB*	In our laboratory
pRE112-4052	*AHA_4052* knockout vector	In this study
pBBRMCS1	Cloning vector, Cm^r^	In our laboratory
pBBR1-4052	*AHA_4052* complement vector	In this study

### Construction of the *AHA_4052* mutant and complemented strains

The *AHA_4052* gene deletion strain was constructed using the suicide vector pRE112 and the homologous recombination principle (Li et al., 2018). The primers are listed in Supplementary Table 1. Briefly, ~500 bp upstream and downstream regions flanking the *AHA_4052* gene were amplified from *A. hydrophila* genomic DNA and fused to the pRE112 plasmid. The recombinant plasmid was transformed into *E. coli* MC1061 competent cells. Verified positive plasmids were subsequently transferred to *E. coli* S17-1 cells. Conjugation between *E. coli* S17-1 (harboring the pRE112 recombinant vector) and wild-type *A. hydrophila* was performed at a 4:1 ratio to facilitate the first homologous recombination, integrating the vector into the *A. hydrophila* genome. This process was screened on an LB agar plate containing ampicillin (Amp) and chloramphenicol (Cm). Then, the second homologous recombination was completed by screening in LB medium supplemented with 20% sucrose. PCR and DNA sequencing were performed on single clones that are sensitive on the Cm plates. Finally, after approximately 20 generations of stable inheritance, the successfully verified *ΔAHA_4052* strain was stored at −80 °C for subsequent experiments.

The complemented *ΔAHA_4052* strain was constructed using the plasmid pBBRMCS1. The full-length *AHA_4052* gene with its promoter region and 6 × His tagged sequence was amplified from *A. hydrophila* genomic DNA, ligated into the pBBRMCS1 vector, and then transformed into *E. coli* competent cells. The plasmids that were completely correct in PCR and sequencing, along with the empty vector pBBR1MCS1, were extracted and transferred to *ΔAHA_4052* competent cells by electro-transformation, and the complemented strain *ΔAHA_4052* + *AHA_4052* was verified by PCR and sequencing again.

### Bacterial tolerance assay

*Aeromonas hydrophila* wild-type and *ΔAHA_4052* mutant strains were cultured overnight at 30 °C, and then the bacteria were transferred at 1% and sub-packed into a HONEYCOMB® Sterile 100-well plate, with 350 μL per well. The OD_600_ nm value was determined using a Bioscreen C instrument (Oy Growth Curves AB Ltd., Helsinki, Finland). Parameters were set to record the data every 2 h over 16 h at 30 °C. For high temperature stress, the instrument temperature was set at 42 °C; for osmotic pressure stress, 4% NaCl was added when transferring bacteria, and for acidic stress, the pH of LB medium was adjusted to 4.9. Finally, the results were generated using GraphPad Prism 10 software.

### Minimum bactericidal concentration determination

Minimum bactericidal concentrations (MBCs) were determined by the agar dilution method (Li et al., 2021). LB agar plates of antibiotics with two-fold serial dilution gradient concentrations were self-prepared, including the following antibiotics: tobramycin (TOB), apramycin (APR), kanamycin (KAN), gentamicin (GEN), streptomycin (STR), paromomycin (PAR), neomycin (NEO), amikacin (AMK), doxycycline (DOX), oxytetracycline hydrochloride (OXY), methyltetracycline hydrochloride (MT), tetracycline (TET), ciprofloxacin (CIP), levofloxacin (LVX), pefloxacin (PEF), enrofloxacin (ENR), moxifloxacin (MFX), enoxacin (ENO), norfloxacin (NOR), ceftriaxone sodium (CRO), moxalactam (MOX), cefamandole (MAN), cefmetazole sodium (CMZ), meropenem (MEM), aztreonam (AZT), azithromycin (AZM), lincomycin (LIN), roxithromycin (ROX), chloramphenicol (CHL), rifampicin (RIF), vancomycin (VAN), trimethoprim (TMP), and natamycin (NAT). The wild-type and *ΔAHA_4052* strains were cultured to an OD_600_ nm of approximately 1.0 and diluted 100-fold, and then, *2-μl* diluted bacterial suspensions were taken and spotted onto the antibiotic-containing LB plate. After 14–16 h incubation at 30 °C, MBC results were recorded. Experiments were repeated independently at least three times. Subsequently, the MBCs for aminoglycoside antibiotics (tobramycin, apramycin, kanamycin, gentamicin, streptomycin, paromomycin, neomycin, and amikacin) were further tested against wild-type, *ΔAHA_4052, ΔAHA_4052* + *AHA_4052*, and *ΔAHA_4052* + vector strains.

### Preparation and trypsin digestion of bacterial whole protein samples

*Aeromonas hydrophila* wild-type and *ΔAHA_4052* mutant strains were cultured overnight at 30 °C, and the bacteria were diluted 1% into 50 mL of fresh LB medium. After growing to OD600_nm_ ≈ 1.0, the cells were pelleted by centrifugation for 20 min at 8,000 g at 4 °C and washed twice with pre-cooled phosphate-buffered saline (PBS). The pellets were resuspended in 1 mL of lysis buffer (6 M urea, 2 M thiourea, 100 mM Tris–HCl [pH 7.6], and protease inhibitor). Next, the ice was subjected to ultrasonic crushing for 15 minutes at 30% power, in 6-second bursts followed by 9-second breaks, until the sample was clear and transparent. Finally, the supernatants were collected by centrifugation for 15 min at 18,000 g at 4 °C, and protein concentrations were performed using the bicinchoninic acid (BCA) assay. 50 μg mixture sample proteins was prepared for building spectral library using the filter-aided sample preparation (FASP) method and trypsin for digestion (Ni et al., 2017), and then, the peptides were dried in a CentriVap concentrator (Labconco Inc., Kansas City, MO, United States).

### Data-independent acquisition (DIA) quantitative proteomics based on spectral library

Peptides from the mixture samples were dissolved in a buffer containing 2% acetonitrile and 0.1% formic acid (pH = 10) and separated using a RIGOL L-3000 high-performance liquid chromatography (HPLC) system (Puyuanjingdian Science and Technology, Ltd., Beijing, China) with a Gemini-NX C18 110A column (size of 250 × 4.6 mm, particle size of 5 μm, Phenomenex, USA) at 1 mL/min using mobile phases A (2% ACN, pH = 10) and B (98% ACN, pH = 10) with a gradient of 5–30% B for 25 min. Ten fractions were obtained and dried under vacuum.

The fractioned and sample peptides were analyzed on the Orbitrap Fusion Lumos system with the same LC condition; a specific process was performed as described previously (Wang et al., 2025a). Solvent A for LC was composed of 0.1% formic acid in water, while solvent B contained 80% acetonitrile and 0.1% formic acid in water. Lyophilized peptides were dissolved in 0.1% formic acid in water and then centrifuged for 15 min at 15,000 g. The resulting supernatant was injected into a C18 analytical column (150 um × 25 cm) using an EASY-nLC 1,200 HPLC (Thermo Fischer Scientific, USA) at a maximum pressure of 300 bar with 12 μL Solvent A. Peptides were eluted from the analytical column at a flow rate of 600 nL/min with a gradient to 6% B at 0 min, 12% B at 18 min, 20% B at 77 min, 32% B at 109 min, and 80% B at 110 min, holding until 120 min.

The fractionated peptides were scanned by the Data-Dependent Acquisition (DDA) model for the spectral library. The eluted peptide was sprayed at a voltage of 2.0 kV with a Nanospray Flex ion source. The ion transfer tube was set at 320 °C. The MS scan resolution was 60,000, the scanning range was 300–1,400 m/z, and the maximum injection time was set to 50 ms. The MS/MS scan resolution was 30,000, using 30 scanning windows, with the first mass set at 120 m/z, the collision energy of 30%, the Automatic gain control (AGC) target of 5e4, and the maximum injection time of 54 ms.

The sample peptides were scanned by the DIA model for sample quantification. The eluted peptide was sprayed at a voltage of 2.0 kV with a Nanospray Flex ion source. The ion transfer tube was set at 320 °C. The MS scan resolution was 60,000, the scanning range was 425–925 m/z, and the maximum injection time was set to 50 ms. The MS/MS scan resolution was 30,000, using a scanning range for MS/MS at 200–1800 m/z, with 45 DIA windows, the collision energy of 33%, the AGC target of 5e5, and the maximum injection time of 60 ms.

DDA raw data were processed using Spectronaut Pulsar 16 software for building a spectral library against the Uniprot *A. hydrophila* ATCC7966 database with the default parameter. For the DIA data, default parameters were used, and protein identification was performed with a precursor, peptide, and protein false discovery rate (FDR) *Q*-value cutoff of 0.01 (Wang et al., 2025b). Furthermore, the protein abundance ratio of the *ΔAHA_4052* strain was calculated, and a *t*-test was performed on the raw data with *A. hydrophila* wild-type as the control. Proteins with the number of peptides greater than 2 and abundance ratio fold change (FC) of ≥2 or ≤0.5 and a *p*-value of <0.05 were classified as the differentially expressed proteins for further analysis. The mass spectrometry proteomic data have been deposited to the ProteomeXchange Consortium^1^ via the iProX partner repository with the dataset identifier PXD067330.

### Bioinformatics analysis

The differentially expressed proteins were subjected to Gene Ontology (GO) and Kyoto Encyclopedia of Genes and Genomes (KEGG) enrichment using OmicsBean software^2^. The results were visualized using Graphpad Prism 10 software and the R GOplot package. Protein–protein interaction networks were analyzed using STRING software, and visualized graphics were generated using Cytoscape software. Finally, antibiotic resistance-associated proteins were predicted using the Comprehensive Antibiotic Research Database (CARD2).

### Chromatin immunoprecipitation assay and PCR validation

Chromatin immunoprecipitation assay (ChIP) is ideally suited for studying protein–DNA interactions *in vivo*; this study used ChIP to investigate the regulation between transcriptional regulation factor *AHA_4052* and candidate genes. When *ΔAHA_4052* + *AHA_4052* and *ΔAHA_4052* + vector strains were cultured to an OD600_nm_ of approximately 1.0, the cells were collected by centrifugation for 10 min at 6,000 g at 4 °C and washed twice with pre-cooled PBS. The cells were cross-linked with 1% formaldehyde for 5 min, sheared to approximately 300–500 bp by sonication on ice for 20 min at 30% power with intervals of 9 s, and subjected to immunoprecipitation with BeaverBeads™ IDA-Nickel (His-tag) for 4–6 h at 4 °C. Elution of DNA from precipitated immunocomplexes was performed using elution buffer (containing 20 mM PBS, 500 mM NaCl, and 500 mM Imidazole, pH = 7.4). Then, 40 μL of 10% SDS and 2 μL of 10 mg/mL protease K were added and incubated overnight at 37 °C. The DNA was extracted with phenol:chloroform:isoamyl alcohol (25:24:1) solution, and then, PCR was performed with specific primers (Supplementary Table 2).

## Results

### The *AHA_4052* mutant affects high temperature and high osmotic pressure in *A. hydrophila*

**Figure 1:**
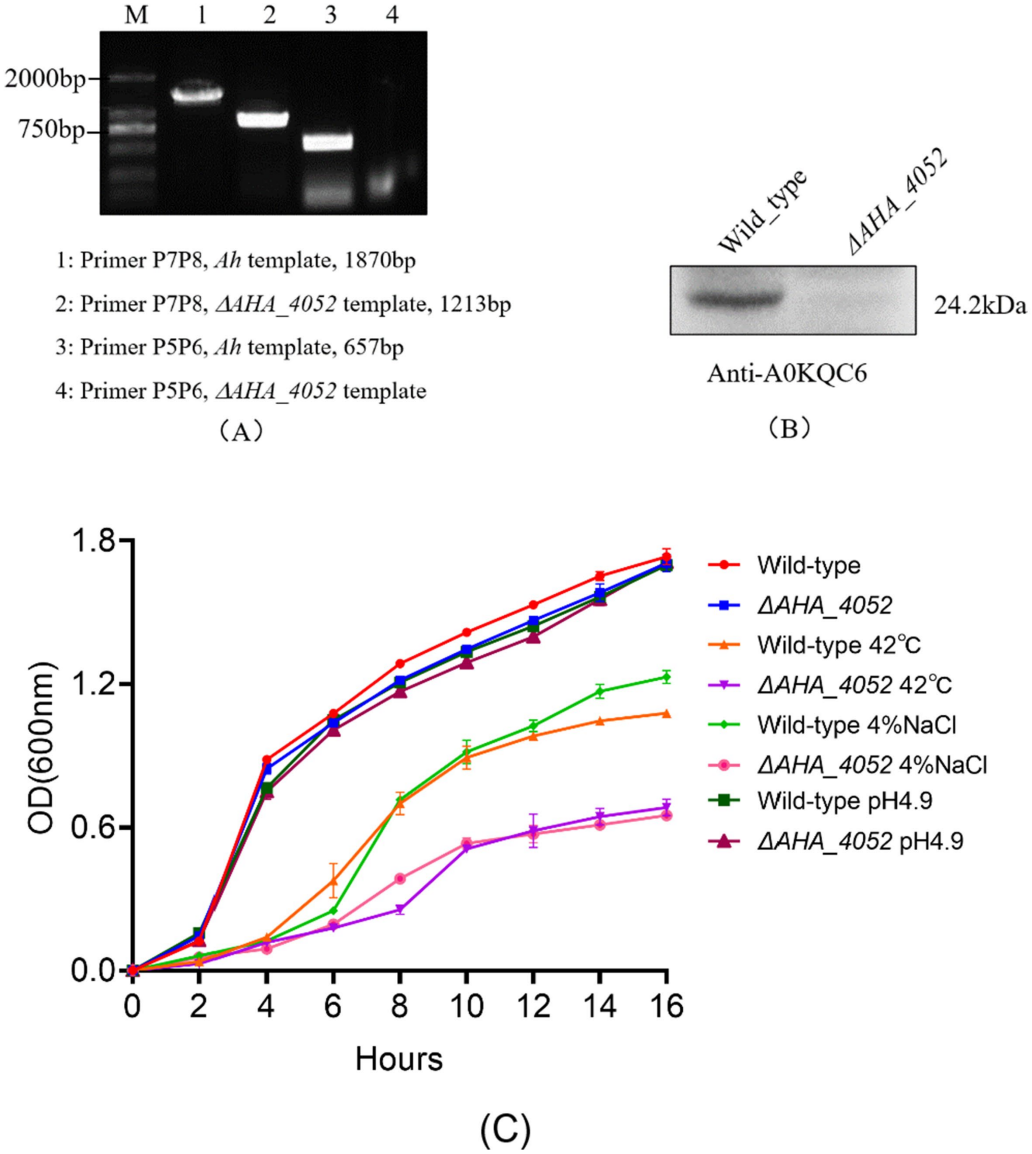
Validation of the *ΔAHA_4052* mutant strain and its stress tolerance assays. **(A)** PCR and **(B)** Western blotting validation of the *ΔAHA_4052* strain. **(C)** Growth curves of *ΔAHA_4052* and wild-type strains under high temperature, high osmotic pressure, and acid (pH 4.9) environmental stress.

To gain a more comprehensive understanding of the biological function of the *AHA_4052* gene, we successfully constructed a *ΔAHA_4052* mutant in *A. hydrophila* using suicide vector pRE112 and homologous recombination methods. The PCR and Western blot results showed no bands at the target location (Figures 1A,B), and further sequencing analyses confirmed the successful knockout of the *AHA-4052* gene. The growth state and tolerance to environmental stress (high temperature, osmosis, and acid) of *ΔAHA_4052* strain were further tested. The results showed that there was no significant difference in the growth rate and acid tolerance between the *ΔAHA_4052* mutant and wild-type strain, indicating that the *AHA_4052* gene is not essential in regulating the survival and adaptation to acidic environments of *A. hydrophila*. However, the *ΔAHA_4052* mutant exhibited significantly reduced tolerance to high temperature (42 °C) and high osmotic pressure (4% NaCl) compared to the wild-type strain (Figure 1C).

### *AHA_4052* is involved in aminoglycoside antibiotic susceptibility of *A. hydrophila*

**Figure 2:**
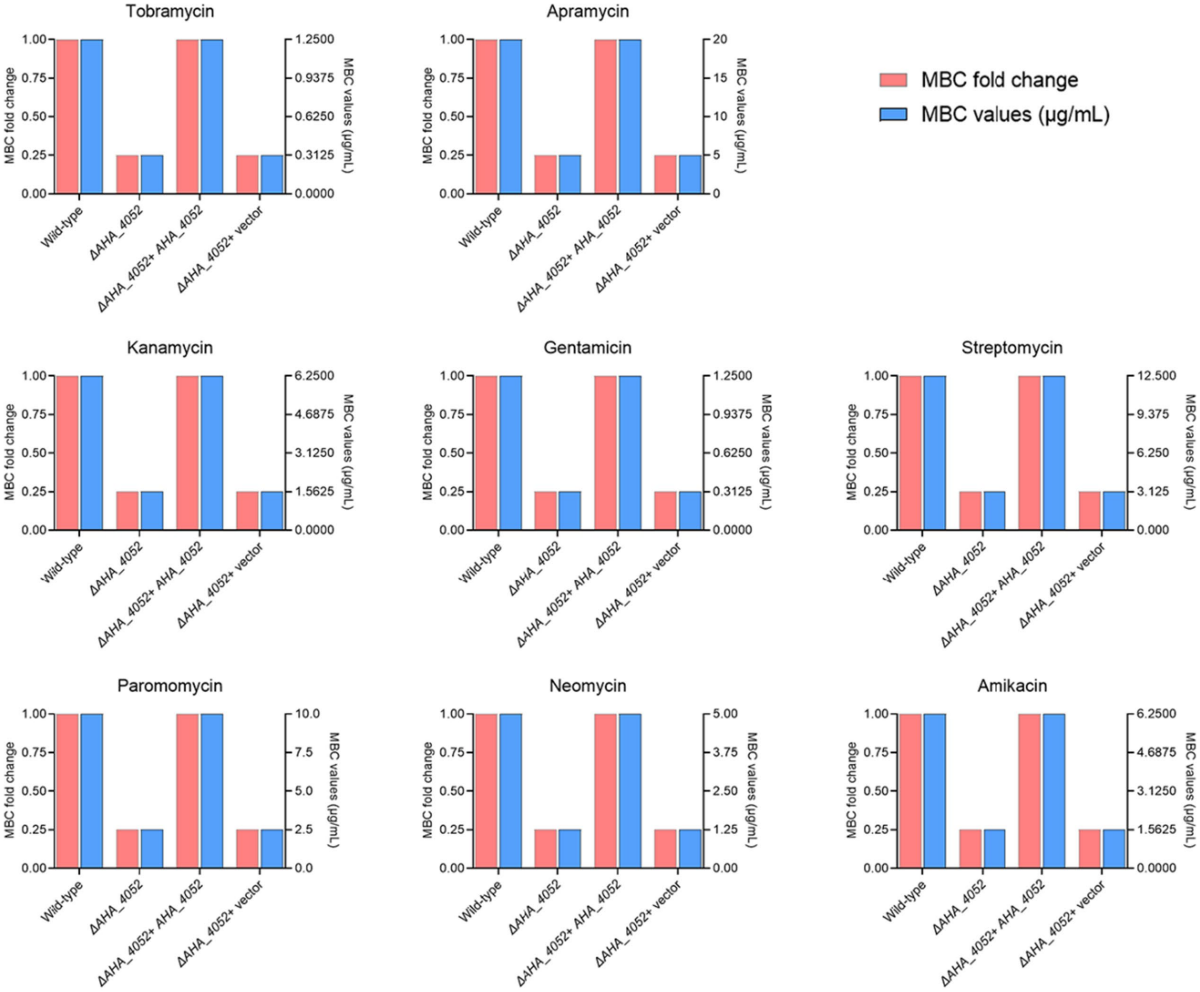
Minimum bactericidal concentrations (MBCs) of eight aminoglycoside antibiotics against wild-type and *ΔAHA_4052* mutant strains of *A. hydrophila*. MBC values of the *ΔAHA_4052* strain for each antibiotic are expressed as fold change relative to the wild-type strain (1 × MBC).

To investigate whether the transcriptional regulation factor *AHA_4052* is involved in regulating antibiotic resistance in *A. hydrophila*, we tested the *ΔAHA_4052* mutants against 33 antibiotics spanning six classes: aminoglycosides including TOB, APR, KAN, GEN, STR, PAR, NEO, and AMK; tetracyclines including DOX, OXY, MT, and TET; quinolones including CIP, LVX, PEF, ENR, MFX, ENX, and NOR; cephalosporins including CRO, MOX, MAN, and CMZ; β - lactams including MEM and AZT, macrolides including AZM and ROX, and other antibiotics including LIN, CHL, RIF, VAN, TMP, and NAT (Supplementary Figure 1). The results showed that the *ΔAHA_4052* mutant exhibited a 4-fold reduction in MBC values for all aminoglycoside antibiotics compared to the wild-type strain while showing no significant differences in susceptibility to other antibiotic classes. The MBC of its complementary strain against aminoglycoside antibiotics was further determined, and it was found that complementation of the mutant restored aminoglycoside MBCs to wild-type levels (Figure 2), demonstrating that the transcriptional regulation factor *AHA_4052* is essential for intrinsic aminoglycoside resistance in *A. hydrophila*.

### Proteomic analysis of differential protein expression between WT and *ΔAHA_4052* strains

**Figure 3:**
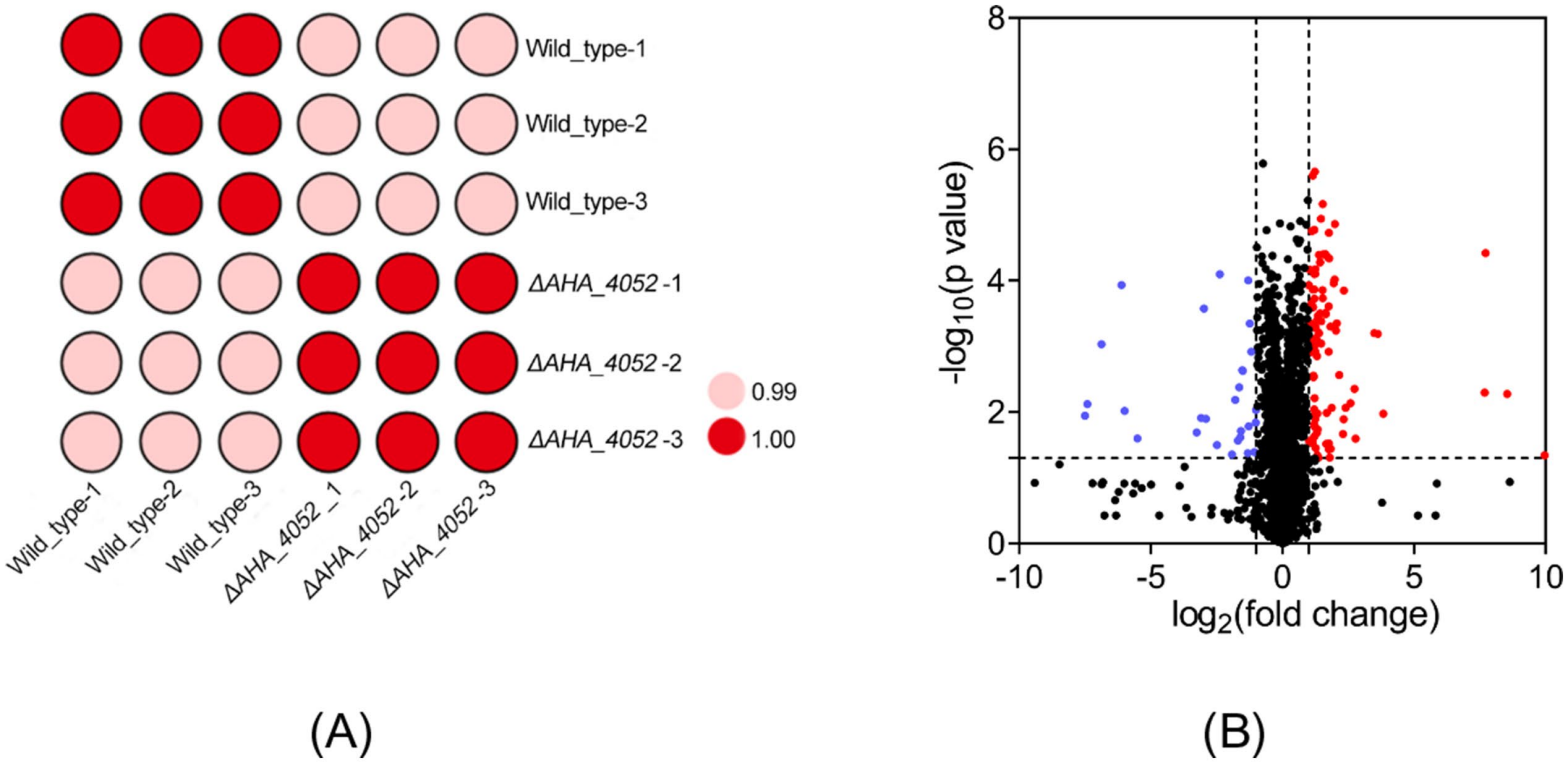
Proteomic profiling of the *ΔAHA_4052* mutant. (A) Correlation analysis of protein intensities between *A. hydrophila* wild-type and *ΔAHA_4052* strains. **(B)** Volcano plots of differentially expressed proteins (|fold change| ≥ 2 and *p*-value <0.05). Each dot represents one protein; a blue dot represents downregulated proteins, and a red dot represents upregulated proteins.

To elucidate the regulatory role of the transcriptional regulation factor *AHA_4052* in directly or indirectly modulating bacterial biological functions, we collected whole protein samples of wild-type and *ΔAHA_4052* strains for quantitative detection using LC–MS/MS technology. LC–MS/MS analysis identified 2,654 proteins with a considerable conservative threshold, including a confidence level ≥95%, at least two unique peptide matches, and a false discovery rate (FDR) of <1% (Supplementary Table 3). A correlation analysis showed that there was a very high correlation between the three biological repeats of protein quantitative data from wild-type and *ΔAHA_4052* samples, and the regression coefficient was greater than 0.99 (Figure 3A). In this study, compared with the wild-type strain, a total of 131 proteins in the *ΔAHA_4052* mutant were significantly differentially expressed (fold change ≥2 or ≤0.5 and *p*-value < 0.05), of which 28 proteins were downregulated and 103 proteins were upregulated (Figure 3B).

### Bioinformatic analysis of differentially expressed proteins between WT and *ΔAHA_4052* strains

**Figure 4:**
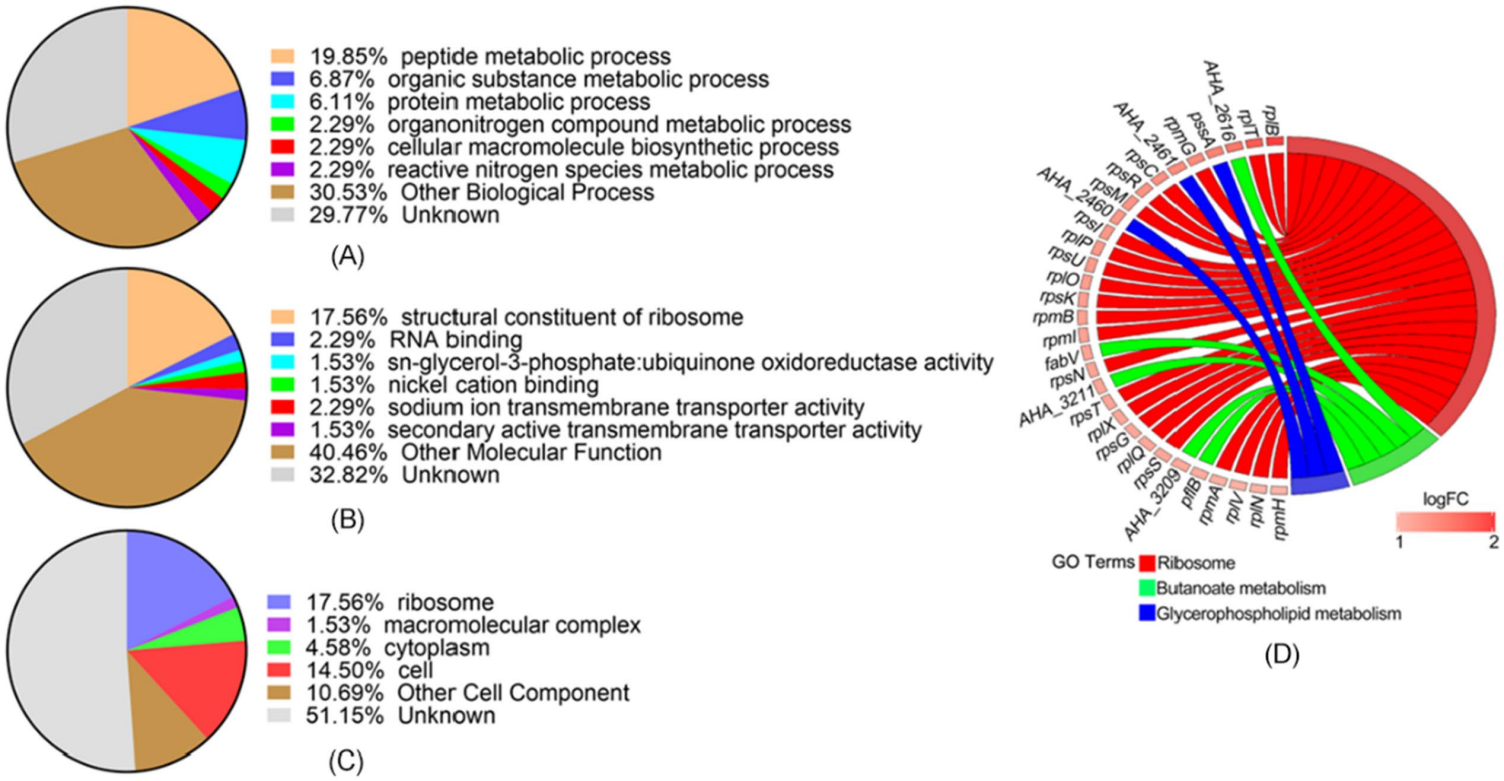
Functional annotation of differentially expressed proteins in the *ΔAHA_4052* strain. Gene Ontology analysis of **(A)** biological processes, **(B)** molecular functions, and (C) cellular components. **(D)** The chord plot showed KEGG pathway enrichment analysis in the *ΔAHA_4052* strain. The left color bar indicates the trend of protein expression, red represents up-expression genes, and blue represents down-expression. Colored bands link genes to corresponding KEGG terms.

We conducted GO functional classification and enrichment analysis of differentially expressed proteins in the *ΔAHA_4052* mutant. In the *ΔAHA_4052* mutant, the top three biological processes were identified as the peptide metabolic process (19.85%), the organic substance metabolic process (6.87%), and the protein metabolic process (6.11%) (Figure 4A). The molecular function category was predominantly represented by structural constituents of ribosomes (17.56%) (Figure 4B), while the primary cellular components were ribosomes (17.56%), cells (14.5%), and cytoplasm (4.58%) (Figure 4C). Notably, a substantial proportion of proteins remained unclassified across all categories: biological processes (29.77%), molecular functions (32.82%), and cellular components (51.15%), indicating undefined functional roles. KEGG metabolic pathway analysis revealed significant enrichment in ribosome, butanoate metabolism, and glycerophospholipid metabolism, with corresponding proteins showing marked upregulation (Figure 4D).

### Protein–protein interaction network analysis of differentially expressed proteins between WT and *ΔAHA_4052* strains

**Figure 5:**
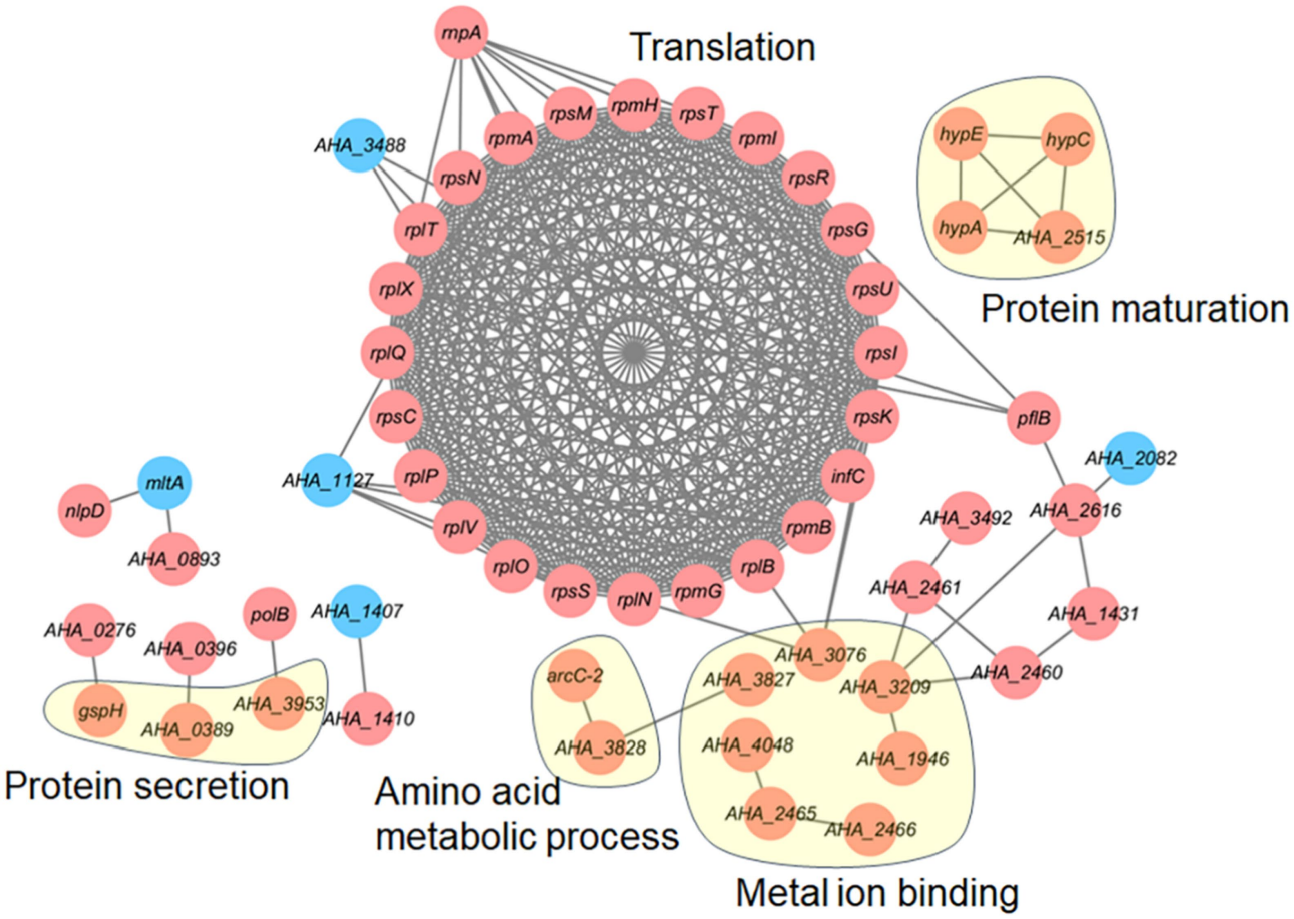
Protein–protein interaction network of differentially expressed proteins in the *ΔAHA_4052* strain. The red circle represents upregulated proteins, and the blue circle represents downregulated proteins.

We further investigated the protein–protein interaction (PPI) network of the differentially expressed proteins in the *ΔAHA_4052* mutant. As shown in Figure 5, numerous altered proteins formed a complex PPI network, with 24 translation-related proteins demonstrating significant enrichment and substantial upregulation. Other key functional clusters included seven metal ion-binding proteins (*AHA_3076*, *AHA_3827*, *AHA_4048*, *AHA_2465*, *AHA-2466*, *AHA-1946,* and *AHA_3209*), four proteins involved in protein maturation (*hypA*, *hypC*, *hypE*, and *AHA_2515*), three components of the protein secretion system (*gspH*, *AHA_0389*, and *AHA_3953*), and two amino acid metabolism-related enzymes (*arcC-2* and *AHA_3828*), and all identified proteins exhibited elevated expression levels. These findings suggest that the transcriptional regulation factor *AHA_4052* modulates multiple bacterial metabolic pathways.

### Role of drug resistance gene in the *ΔAHA_4052* mutant

To investigate the antibiotic resistance mechanism of the transcriptional regulation factor *AHA_4052*, we identified differentially expressed antibiotic resistance genes (ARGs) in the *ΔAHA_4052* mutant using the Comprehensive Antibiotic Research Database (CARD). Seven resistance genes were found to be directly or indirectly regulated by the transcriptional regulation factor *AHA_4052*, with five proteins (*aheB*, *AHA_0484*, *selB*, *AHA_3076,* and *AHA_0232*) showing increased abundance and two proteins (*AHA_2114* and *AHA_3488*) exhibiting decreased abundance. These proteins mediate bacterial antibiotic resistance through mechanisms including efflux pumps, antibiotic inactivation, target alteration, and target protection (Table 2).

**Table 2:** Resistance genes in the *ΔAHA_4052* mutant.

Accessions	Genes	Descriptions	Ratio	*T*-test	Drug resistance function
A0KL71	*selB*	Selenocysteine-specific translation elongation factor	3.12	0.0003	Antibiotic efflux
A0KFJ1	*AHA_0484*	Membrane-fusion protein	2.23	0.0300	Antibiotic efflux
A0KMB3	*aheB*	Efflux pump membrane transporter	2.33	0.0061	Antibiotic efflux and target alteration
A0KMS7	*AHA_3076*	Molybdenum transport ATP-binding protein ModC	3.16	0.0304	Antibiotic efflux, inactivation, target alteration, and target protection
A0KEU4	*AHA_0232*	LysR family transcriptional regulator	3.56	0.0362	Antibiotic efflux
A0KK37	*AHA_2114*	Multidrug-resistance protein MdtK	0.48	0.0404	Antibiotic efflux and target protection
A0KNW1	*AHA_3488*	ABC transporter, ATP-binding protein	0.44	0.0012	Antibiotic efflux and target protection

**Figure 6:**
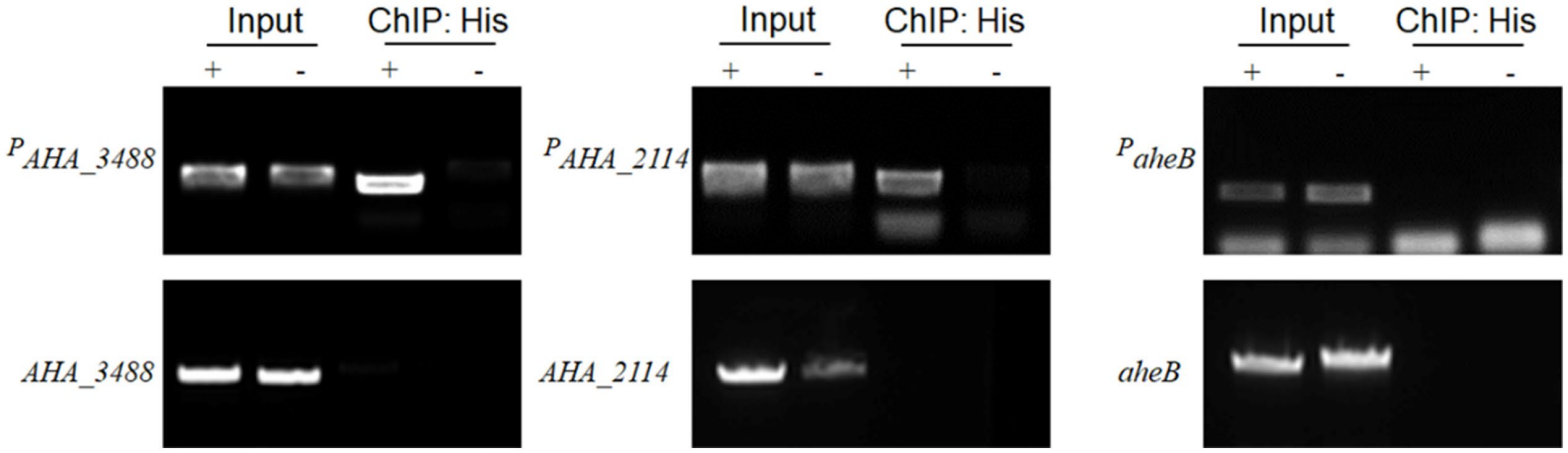
The chromatin immunoprecipitation and PCR assay of transcriptional regulation factor *AHA_4052* with the promoters of *AHA_3488*, *AHA_2114*, and *aheB* genes. *ΔAHA_4052* + *AHA_4052* complementary strain was marked as “+”; *ΔAHA_4052* + vector strain was marked as “–.” The chromatin of *ΔAHA_4052* + *AHA_4052* and *ΔAHA_4052* + vector strains was immunoprecipitated with or without anti-His antibody (input or ChIP:His) and validated by PCR. The promoter fragment of the gene is labeled *^P^gene name*.

Subsequently, to further verify whether transcriptional regulation factor *AHA_4052* directly or indirectly regulates drug resistance genes (*AHA_3488*, *AHA_2114,* and *aheB*), we used chromatin immunoprecipitation assay and PCR technology (ChIP-PCR) to determine the binding capability of candidate genes with *AHA_4052*. The results showed that three genes could be amplified in the *ΔAHA_4052* + *AHA_4052* and *ΔAHA_4052* + vector input samples. In addition, the predicted promoter regions upstream of the *AHA_3488* and *AHA_2114* genes (*^P^AHA_3488* and *^P^AHA_2114*) could be amplified in the ChIP sample of the *ΔAHA_4052* + *AHA_4052* strain, while their own gene fragments could not be amplified, and neither fragment could be amplified in the *ΔAHA_4052* + vector ChIP sample. At the same time, the predicted promoter region of the *aheB* gene and its gene fragment were not amplified in ChIP samples (Figure 6). These results demonstrate that the transcriptional regulation factor *AHA_4052* may directly regulate drug resistance genes *AHA_3488* and *AHA_2114* by binding their promoters while indirectly regulating *the aheB* gene.

## Discussion

*A. hydrophila* is a prevalent fish pathogen that has increasingly exhibited multidrug resistance. Studies have detected drug-resistant *A. hydrophila* strains have been detected across diverse food sources, posing significant public health concerns (Stratev and Odeyemi, 2016). Although numerous studies have explored the drug resistance mechanism of *A. hydrophila*, the potential drug resistance regulatory network still needs further research.

Many previous studies have established the critical role of the EnvZ/OmpR two-component regulatory system (TCRS) in bacterial adaptation to acid stress, osmotic pressure, and antibiotic resistance through the modulation of outer membrane porins OmpF and OmpC (Ko and Choi, 2022; Wang et al., 2023). Similarly, the PhoP/PhoQ TCRS has been shown to regulate tolerance-related genes and resist various environmental stresses, as well as regulate bacterial resistance to polymyxin B, aminoglycosides, and quinazoline (Macfarlane et al., 2000; Ma et al., 2021; Guo et al., 2022). However, the functional roles of OmpR/PhoB-type proteins in *A. hydrophila*, particularly the transcriptional regulator A0KQC6 (*AHA_4052*) containing an OmpR/PhoB-type DNA-binding domain, remain largely unexplored.

In this study, we constructed the *AHA_4052* gene mutant (*ΔAHA_4052*) and complemented strains in *A. hydrophila* to investigate its biological functions. Subsequently, the growth of the wild-type and *ΔAHA_4052* mutant strains under different environmental stresses (high temperature, osmosis, and acid) and the MBCs of different antibiotics were determined. The results revealed that the *AHA_4052* gene knockout significantly impaired bacterial growth under high-temperature and osmotic stress conditions. Furthermore, the mutant strain exhibited a four-fold reduction in MBCs against aminoglycoside antibiotics. While the observed osmotic regulation aligns with known OmpR/PhoB-type protein functions (Chakraborty and Kenney, 2018), little is known about the regulation of tolerance to temperature and aminoglycoside antibiotics, suggesting that *AHA_4052* may possess novel regulatory functions beyond those previously characterized.

To gain a comprehensive understanding of the mechanisms underlying these phenotypes, we performed label-free quantitative proteomics to analyze the global impact of the *ΔAHA_4052* mutant on protein expression. Proteomic analysis revealed that the *AHA_4052* gene regulates the expression of a range of proteins involved in a wide range of metabolism pathways, including ribosomes, butanoate metabolism, and glycerophospholipid metabolism. In particular, alterations in glycerophospholipid metabolism may affect membrane fluidity and integrity, potentially explaining the mutant’s increased sensitivity to osmotic and heat stress (Wu et al., 2019). Similarly, butanoate metabolism could influence energy homeostasis and stress response signaling pathways, further leading to phenotypic changes (Abdelhamid and Yousef, 2024). However, a substantial proportion of the differentially expressed proteins are of unknown function, indicating that the transcriptional regulator *AHA-4052* may also play a significant role in regulating novel or uncharacterized functions in *A. hydrophila*.

Aminoglycoside antibiotics are concentration-dependent bactericidal agents known for their rapid and potent killing activity. After knocking out the *AHA_4052* gene, the *ΔAHA_4052* mutant significantly reduced the minimum bactericidal concentration of aminoglycosides, and 23 ribosomal proteins, including 13 50S ribosomes and 10 30S ribosomal subunit-related proteins (17.56% of differentially expressed proteins), were significantly upregulated. Previous studies have demonstrated that aminoglycoside antibiotics target the bacterial 30S ribosomal subunit, interfering with the normal ribosomal function by misguiding translation to synthesize non-functional or toxic proteins and by blocking translation, leading to a complete halt in protein synthesis, thereby exerting their bactericidal effect (Dunkle et al., 2014; Wang N. Y. et al., 2022). Therefore, we propose a model wherein the upregulation of ribosomal proteins upon *AHA_4052* gene deletion increases the number of potential binding sites for aminoglycosides within the cell. This could facilitate more rapid and extensive binding of the antibiotics, thereby potentiating their bactericidal effect by exacerbating translational errors and inhibition. In contrast, the resistance levels to other antibiotic classes such as tetracyclines, quinolones, and cephalosporins remained unchanged in the mutant, which may be attributed to the specificity of drug targets and efflux systems; for instance, aminoglycoside efficacy is closely tied to ribosomal interaction, whereas other antibiotics often depend on distinct mechanisms such as cell wall synthesis or DNA replication, which appear unaffected by *AHA_4052* deletion. In summary, these findings imply that the *AHA_4052* gene in *A. hydrophila* may modulate bacterial resistance to aminoglycosides through the regulation of ribosomal protein expression.

Beyond the potential indirect effect mediated by ribosomal upregulation, we aimed to identify direct targets of *AHA_4052* that are known to be associated with antibiotic resistance. Comprehensive Antibiotic Resistance Database (CARD) analysis identified seven resistance-associated proteins with altered expression in *ΔAHA_4052*, revealing a complex regulatory network impacting drug resistance. In this study, the downregulation of A0KK37 (*AHA_2114*, multidrug-resistant protein, MATE family MdtK) and A0KNW1 (*AHA_3488*, adenosine triphosphate (ATP)-binding cassette (ABC) transporter, ATP-binding protein) in *ΔAHA_4052* indicates a direct impairment of critical efflux systems, likely contributing to a significant decrease in antibiotic resistance in the mutants. Studies have reported that the MATE family is a group of multidrug-resistant efflux proteins closely associated with antibiotic resistance. For example, the expression of MATE family proteins in *Staphylococcus aureus* confers the bacterium resistance to norfloxacin and ciprofloxacin (Kaatz et al., 2005; Claxton et al., 2021). In *A. hydrophila*, knocking out the *AHA_2144* gene (a MATE homolog), the bacteria are sensitive to enoxacin, indicating that this gene plays a certain role in bacterial resistance (Li et al., 2021). ABC transporters are involved in the influx or efflux of various molecules, which is a key part of the mechanism of antibiotic resistance. In bacteria, ABC transporters are found to be involved in the resistance of daunorubicin, doxorubicin, macrolides, colistin, and other antibiotics (Hürlimann et al., 2016; Orelle et al., 2019). Furthermore, chromatin immunoprecipitation-PCR (ChIP-PCR) confirmed direct regulatory interactions between transcriptional regulation factor *AHA_4052* and the resistance genes *AHA_2114* and *AHA_3488*. Conversely, the upregulation of proteins A0KFJ1 (*AHA_0484*, membrane-fusion protein) and A0KMB3 (*aheB*, efflux pump membrane transporter) may represent a compensatory adaptation to the loss of the primary pumps. Meanwhile, the concurrent upregulation of other resistance factors such as A0KL71 (*selB*, selenocysteine-specific translation elongation factor), whose increase implies enhanced selenocysteine incorporation potentially boosting antioxidant defenses to mitigate antibiotic-induced oxidative stress (Mukai, 2021), as well as A0KMS7 (*AHA_3076*, molybdenum transport ATP-binding protein ModC) and A0KEU4 (*AHA_0232*, LysR family transcriptional regulator), suggests that *AHA_4052* influences global stress responses and metabolic pathways linked to antibiotic efficacy. Collectively, these findings indicate that *AHA_4052* acts as a master regulator, potentially directly regulating key efflux pumps and indirectly modulating broader resistance that includes oxidative stress management and metabolic adaptation, thereby integratively shaping the antibiotic resistance spectrum of *A. hydrophila*.

## Conclusion

This study demonstrates that the transcriptional regulator *AHA_4052* in *A. hydrophila* plays a critical role in bacterial adaptation to thermal stress, osmotic pressure, and aminoglycoside resistance. Proteomic and molecular validation revealed that *AHA_4052* exerts a dual regulatory function: it indirectly modulates aminoglycoside susceptibility through ribosomal protein expression, and directly controls the transcription of efflux pumps (*AHA_2114*) and ABC transporters (*AHA_3488*). Therefore, it will be very interesting to further study the mechanism of the transcriptional regulation factor *AHA_4052* in aminoglycoside antibiotic resistance of *A. hydrophila* and to explore whether interfering with its mechanism can effectively kill the bacteria with low-dose antibiotics.

## Data Availability

The datasets presented in this study can be found in online repositories. The names of the repository/repositories and accession number(s) can be found in the article/Supplementary material.

## Footnotes

^1^ https://proteomecentral.proteomexchange.org

^2^ http://www.omicsbean.cn/

## References

[ref1] Abdelhamid A. G. Yousef A. (2024). Untargeted metabolomics unveiled the role of butanoate metabolism in the development of *Pseudomonas aeruginosa* hypoxic biofilm. Front. Cell. Infect. Microbiol. 14:1346813. doi: 10.3389/fcimb.2024.1346813, PMID: 38435305 PMC10904581

[ref2] Borella L. Salogni C. Vitale N. Scali F. Moretti V. M. Pasquali P. . (2020). Motile aeromonads from farmed and wild freshwater fish in northern Italy: an evaluation of antimicrobial activity and multidrug resistance during 2013 and 2016. Acta Vet. Scand. 62, 6–8. doi: 10.1186/s13028-020-0504-y, PMID: 31973764 PMC6979286

[ref3] Cabezudo I. Lobertti C. A. Garcia Vescovi E. Furlan R. L. E. (2022). Effect-directed synthesis of PhoP/PhoQ inhibitors to regulate *Salmonella* virulence. J. Agric. Food Chem. 70, 6755–6763. doi: 10.1021/acs.jafc.2c01087, PMID: 35607919

[ref4] Cai S. J. Inouye M. (2002). EnvZ-OmpR interaction and osmoregulation in *Escherichia coli*. J. Biol. Chem. 277, 24155–24161. doi: 10.1074/jbc.M110715200, PMID: 11973328

[ref5] Cao Q. Yang N. Wang Y. H. Xu C. C. Zhang X. Fan K. . (2020). Mutation-induced remodeling of the BfmRS two-component system in *Pseudomonas aeruginosa* clinical isolates. Sci. Signal. 13:eaaz1529. doi: 10.1126/scisignal.aaz1529, PMID: 33144518

[ref6] Chakraborty S. Kenney L. J. (2018). A new role of OmpR in acid and osmotic stress in *Salmonella* and *E. coli*. Front. Microbiol. 9:2656. doi: 10.3389/fmicb.2018.02656, PMID: 30524381 PMC6262077

[ref7] Chen H. B. Xiong Z. J. Liu K. Y. Li S. G. Wang R. B. Wang X. J. . (2016). Transcriptional profiling of the two-component regulatory system VraSR in *Staphylococcus aureus* with low-level vancomycin resistance. Int. J. Antimicrob. Agents 47, 362–367. doi: 10.1016/j.ijantimicag.2016.02.003, PMID: 27084050

[ref8] Claxton D. P. Jagessar K. L. Mchaourab H. S. (2021). Principles of alternating access in multidrug and toxin extrusion (MTE) transporters. J. Mol. Biol. 433:166959. doi: 10.1016/j.jmb.2021.166959 33774036 PMC8292195

[ref9] Dahdouh B. Basha O. Khalil S. Tanekhy M. (2016). Molecular characterization, antimicrobial susceptibility and salt tolerance of *Aeromonas hydrophila* from fresh, brackish and marine fishes. Alex. J. Vet. Sci. 48, 46–53. doi: 10.1128/JCM.01099-08

[ref10] De Gaetano G. V. Lentini G. Famà A. Coppolino F. Beninati C. (2023). Antimicrobial resistance: two-component regulatory systems and multidrug efflux pumps. Antibiotics (Basel) 12:965. doi: 10.3390/antibiotics12060965, PMID: 37370284 PMC10295115

[ref11] De Silva L. A. D. S. Heo G.-J. (2022). Biofilm formation of pathogenic bacteria isolated from aquatic animals. Arch. Microbiol. 205:36. doi: 10.1007/s00203-022-03332-8, PMID: 36565346

[ref12] Dunkle J. A. Vinal K. Desai P. M. Zelinskaya N. Savic M. West D. M. . (2014). Molecular recognition and modification of the 30S ribosome by the aminoglycoside-resistance methyltransferase NpmA. Proc. Natl. Acad. Sci. U. S. A. 111, 6275–6280. doi: 10.1073/pnas.1402789111, PMID: 24717845 PMC4035980

[ref13] Fernandez-Ciruelos B. Potmis T. Solomin V. Wells J. M. (2023). Cross-talk between QseBC and PmrAB two-component systems is crucial for regulation of motility and colistin resistance in enteropathogenic *Escherichia coli*. PLoS Pathog. 19:e1011345. doi: 10.1371/journal.ppat.1011345, PMID: 38060591 PMC10729948

[ref14] Gao R. Mack T. R. Stock A. M. (2007). Bacterial response regulators: versatile regulatory strategies from common domains. Trends Biochem. Sci. 32, 225–234. doi: 10.1016/j.tibs.2007.03.002, PMID: 17433693 PMC3655528

[ref15] Groisman E. A. Duprey A. Choi J. J. (2021). How the PhoP/PhoQ system controls virulence and Mg2+ homeostasis: lessons in signal transduction, pathogenesis, physiology, and evolution. Microbiol. Mol. Biol. Rev. 85:e00176-00120. doi: 10.1128/MMBR.00176-20 34191587 PMC8483708

[ref16] Guo H. R. Zhao T. Huang C. Chen J. Y. (2022). The role of the two-component system PhoP/PhoQ in intrinsic resistance of *Yersinia enterocolitica* to polymyxin. Front. Microbiol. 13:758571-758571. doi: 10.3389/fmicb.2022.758571 35222323 PMC8867023

[ref17] Hürlimann L. M. Corradi V. Hohl M. Bloemberg G. V. Tieleman D. P. Seeger M. A. (2016). The heterodimeric ABC transporter EfrCD mediates multidrug efflux in *Enterococcus faecalis*. Antimicrob. Agents Chemother. 60, 5400–5411. doi: 10.1128/AAC.00661-16, PMID: 27381387 PMC4997860

[ref18] Ibrahim M. Ahmad F. Yaqub B. Ramzan A. Imran A. Afzaal M. . (2020). “Chapter 4 -current trends of antimicrobials used in food animals and aquaculture” in Antibiotics and antimicrobial resistance genes in the environment, Ed. M. Lafleur, 39–69. Amsterdam, the Netherlands: Elsevier.

[ref19] Jeamsripong S. Odoi J. O. Shahi M. K. Anuntawirun S. Roongrojmongkhon N. Thiptara A. (2025). Global spread and antimicrobial resistance of *Aeromonas hydrophila* in aquatic food animals: a systematic review and meta-analysis. Sci. Rep. 15:28441. doi: 10.1038/s41598-025-14498-8, PMID: 40759729 PMC12322169

[ref20] Kaatz G. W. McAleese F. Seo S. M. (2005). Multidrug resistance in *Staphylococcus aureus* due to overexpression of a novel multidrug and toxin extrusion (MATE) transport protein. Antimicrob. Agents Chemother. 49, 1857–1864. doi: 10.1128/AAC.49.5.1857-1864.2005, PMID: 15855507 PMC1087643

[ref21] Kaur S. Kaur H. Kaur B. Kumar B. N. Tyagi A. Singh P. . (2024). Isolating pathogenic multidrug-resistant *Aeromonas hydrophila* from diseased fish and assessing the effectiveness of a novel lytic *Aeromonas veronii* bacteriophage (AVP3) for biocontrol. Microb. Pathog. 196:106914. doi: 10.1016/j.micpath.2024.106914 39241817

[ref22] Ko D. Choi S. H. (2022). Mechanistic understanding of antibiotic resistance mediated by EnvZ/OmpR two-component system in *Salmonella enterica* serovar enteritidis. J. Antimicrob. Chemother. 77, 2419–2428. doi: 10.1093/jac/dkac223, PMID: 35781339

[ref23] Li W. X. Ali F. Cai Q. L. Yao Z. J. Sun L. N. Lin W. X. . (2018). Quantitative proteomic analysis reveals that chemotaxis is involved in chlortetracycline resistance of *Aeromonas hydrophila*. J. Proteome 172, 143–151. doi: 10.1016/j.jprot.2017.09.011, PMID: 28986269

[ref24] Li L. F. Ma J. Y. Cheng P. Li M. C. Yu Z. Y. Song X. R. . (2023). Roles of two-component regulatory systems in *Klebsiella pneumoniae*: regulation of virulence, antibiotic resistance, and stress responses. Microbiol. Res. 272:127374. doi: 10.1016/j.micres.2023.127374, PMID: 37031567

[ref25] Li Z. Zhang L. S. Song Q. L. Wang G. B. Yang W. X. Tang H. M. . (2021). Proteomics analysis reveals bacterial antibiotics resistance mechanism mediated by *ahslyA* against Enoxacin in *Aeromonas hydrophila*. Front. Microbiol. 12:699415. doi: 10.3389/fmicb.2021.699415, PMID: 34168639 PMC8217646

[ref26] Long J. F. Xu H. Z. Qi X. Y. Yan C. Y. Sun X. N. Jin Y. J. . (2025). The deletion of the *uvrY* in *Aeromonas veronii* disrupted the BarA/UvrY two-component system, decreasing persister formation and bacterial resistance to multiple antibiotics. Int. J. Food Microbiol. 435:111183. doi: 10.1016/j.ijfoodmicro.2025.111183, PMID: 40168752

[ref27] Ma Y. Zhang Y. Y. Chen K. Zhang L. Z. Zhang Y. B. Wang X. . (2021). The role of PhoP/PhoQ two component system in regulating stress adaptation in *Cronobacter sakazakii*. Food Microbiol. 100:103851. doi: 10.1016/j.fm.2021.103851, PMID: 34416955

[ref28] Macfarlane E. L. A. Kwasnicka A. Hancock R. E. W. (2000). Role of *Pseudomonas aeruginosa* PhoP-PhoQ in resistance to antimicrobial cationic peptides and aminoglycosides. Microbiology (Reading) 146, 2543–2554. doi: 10.1099/00221287-146-10-2543, PMID: 11021929

[ref29] Mao M. Q. He L. Yan Q. P. (2025). An updated overview on the bacterial PhoP/PhoQ two-component signal transduction system. Front. Cell. Infect. Microbiol. 15:1509037. doi: 10.3389/fcimb.2025.1509037, PMID: 39958932 PMC11825808

[ref30] Mukai T. (2021). Bioinformatic prediction of an tRNASec gene nested inside an elongation factor SelB gene in Alphaproteobacteria. Int. J. Mol. Sci. 22:4605. doi: 10.3390/ijms22094605, PMID: 33925673 PMC8124441

[ref31] Ni M. W. Wang L. Chen W. Mou H. Z. Zhou J. Zheng Z. G. (2017). Modified filter-aided sample preparation (FASP) method increases peptide and protein identifications for shotgun proteomics. Rapid Commun. Mass Spectrom. 31, 171–178. doi: 10.1002/rcm.7779, PMID: 27794190

[ref32] Orelle C. Mathieu K. Jault J. M. (2019). Multidrug ABC transporters in bacteria. Res. Microbiol. 170, 381–391. doi: 10.1016/j.resmic.2019.06.001, PMID: 31251973

[ref33] Pessoa R. B. G. Oliveira W. F. D. Correia M. T. D. S. Fontes A. Coelho L. C. B. B. (2022). *Aeromonas* and human health disorders: clinical approaches. Front. Microbiol. 13:1266. doi: 10.3389/fmicb.2022.868890, PMID: 35711774 PMC9195132

[ref34] Stock A. M. Robinson V. L. Goudreau P. N. (2000). Two-component signal transduction. Annu. Rev. Biochem. 69, 183–215. doi: 10.1146/annurev.biochem.69.1.183, PMID: 10966457

[ref35] Stratev D. Odeyemi O. A. (2016). Antimicrobial resistance of *Aeromonas hydrophila* isolated from different food sources: a mini-review. J. Infect. Public Health 9, 535–544. doi: 10.1016/j.jiph.2015.10.006, PMID: 26588876

[ref36] Tierney A. R. Rather P. N. (2019). Roles of two-component regulatory systems in antibiotic resistance. Future Microbiol. 14, 533–552. doi: 10.2217/fmb-2019-0002, PMID: 31066586 PMC6526388

[ref38] Wang G. B. Cao L. Lian L. L. Wang Y. Q. Lian J. Q. Liu Z. Q. . (2025b). Machine learning and DIA proteomics reveal new insights into carbapenem resistance mechanisms in *Klebsiella pneumoniae*. J. Proteome Res. 24, 4002–4014. doi: 10.1021/acs.jproteome.5c00142, PMID: 40622342

[ref37] Wang G. B. Chen L. X. Lian J. Q. Gong L. Q. Tian F. Wang Y. Q. . (2025a). Proteomic insights into the regulatory role of CobQ deacetylase in *Aeromonas hydrophila*. J. Proteome Res. 24, 333–343. doi: 10.1021/acs.jproteome.4c00847, PMID: 39659247

[ref39] Wang Y.-D. Gong J.-S. Guan Y.-C. Zhao Z.-L. Cai Y.-N. Shan X.-F. (2023). OmpR (TCS response regulator) of *Aeromonas veronii* plays a major role in drug resistance, stress resistance and virulence by regulating biofilm formation. Microb. Pathog. 181:106176. doi: 10.1016/j.micpath.2023.106176, PMID: 37244492

[ref40] Wang N. Y. Luo J. Deng F. Huang Y. S. Zhou H. (2022). Antibiotic combination therapy: a strategy to overcome bacterial resistance to aminoglycoside antibiotics. Front. Pharmacol. 13:839808. doi: 10.3389/fphar.2022.839808, PMID: 35281905 PMC8905495

[ref41] Wang T. Tian X.-L. Xu X.-B. Li H. Tian Y. Ma Y.-H. . (2022). Dietary supplementation of probiotics fermented Chinese herbal medicine *Sanguisorba officinalis* cultures enhanced immune response and disease resistance of crucian carp (*Carassius auratus*) against *Aeromonas hydrophila*. Fish Shellfish Immunol. 131, 682–696. doi: 10.1016/j.fsi.2022.10.046, PMID: 36341871

[ref42] Wu C. J. Zhang J. L. Zhu G. X. Yao R. Chen X. L. Liu L. M. (2019). CgHog1-mediated CgRds2 phosphorylation alters glycerophospholipid composition to coordinate osmotic stress in *Candida glabrata*. Appl. Environ. Microbiol. 85:e02822-18. doi: 10.1128/AEM.02822-18, PMID: 30635387 PMC6414385

[ref43] Yang Y. Y. Miao P. F. Li H. Tan S. W. Yu H. Y. Yu H. (2018). Antibiotic susceptibility and molecular characterization of *Aeromonas hydrophila* from grass carp. J. Food Saf. 38:e12393. doi: 10.1111/jfs.12393

[ref44] Zhang B. Y. Zhang Y. Liu J. J. Reverter D. Wang Q. Y. Choi S. H. . (2024). ChIP-seq and structural analyses delineating the regulatory mechanism of master regulator EsrB in *Edwardsiella piscicida*. Appl. Environ. Microbiol. 90:e0180524. doi: 10.1128/aem.01805-24 39545739 PMC11653779

[ref45] Zhu Y. X. Dou Q. Du L. C. Wang Y. (2023). QseB/QseC: a two-component system globally regulating bacterial behaviors. Trends Microbiol. 31, 749–762. doi: 10.1016/j.tim.2023.02.001, PMID: 36849330

