# Supplementary material for: Transcriptional regulatory factor AHA_4052 regulates aminoglycoside resistance in Aeromonas hydrophila
Source: Front Microbiol. 2025 Nov 25;16:1689335. doi: 10.3389/fmicb.2025.1689335 (PMC12685816; doi:10.3389/fmicb.2025.1689335)
Supplement: Supplementary file 1 [file Data_Sheet_1.pdf]

Supplementary Table 1 *AHA_4052* gene knockout primers

Primer	Primer sequence (5'-3')	Size (bp)	Application
P1	cgatcccaagcttcttctagaGCAGGAAACTGCGTCGGAT	511	Amplify the upstream homology arm fragment of
P2	aagagccggcAAATGGGTATCCAAAGCAGGG		*AHA_4052* gene
P3	atacccatttGCCGGCTCTTGATGCCCT	501	Amplify the downstream homology arm fragment of
P4	catgaattcccgggagagctcTGCTGTCCTTGGCCATCACC		*AHA_4052* gene
P5	gccatggctgatatcggatccATGCGAATTCTGATAGTTGAAGATG	657	*AHA_4052* gene verification primer
P6	ctcgagtgcggccgcaagcttTCATTCGTTGATCCGATAGCC		
P7	ggatcttccagagatACGATCTGGAACATGAAACCGA	1870	*AHA_4052* verification primer on the outer side of the
P8	ctgccgttcgacgatCGAATGTCGTGATACCCTTGG		homology arm

Supplementary Table 2 The primers of ChIP

Gene	Primer	Sequence (5 '-3')	Size (bp)
^{P}*aheB*	F	ccagagatGGAGAACCTGAGCCTCTGTCTATC	142
	R	ctgccgttcgacgatCTCTGCGCGCACCTTGTAA	
aheB	F	tccagagatATGGCACGATTTTTCATAGACAGA	3150
	R	gttcgacgatTTAGTCATGTTTGATCTCCTCGGC	
^{P}*AHA_2114*	F	ggatcttccagagatTGACACCTCCGAGTGGATCAG	136
	R	ctgccgttcgacgatGCGCCAAACGTTTCGTCC	
AHA_2114	F	ggatcttccagagatGTGCAACGTTATTGGTCCGAG	1380
	R	ctgccgttcgacgatCTATTGCGCCTTGGAGAGGG	
^{P}*AHA_3488*	F	ggatcttccagagatGACGCGTTTCCTGTTTGCC	169
	R	gccgttcgacgatGATAAGCTGATGTTTACCCCCCT	
AHA_3488	F	tccagagatATGAATTCAACTCCCATTATCAACG	726
	R	ccgttcgacgatTCATGACAGCTCCCTGACTCTTG	
^{P}*AHA_0389*	F	ggatcttccagagatGGTTATCGTGTGGTGGCCAT	151
	R	gttcgacgatCTTGCAATTAAACTCGACAAACCT	
AHA_0389	F	tcttccagagatATGAAAAAACATCATCTCTGCCTG	1674
	R	ctgccgttcgacgatTCAGCCCTTGGGCGGATA	
^{P}*AHA_0396*	F	ctgccgttcgacgatAACCAGCTGAGCTGAGCTGC	182
	R	gagatAGAGATTAAAACTGTCTATGGTTATCCG	
AHA_0396	F	ggatcttccagagatATGCGCAGAGGCTTCACTCTG	492
	R	cgttcgacgatTCAGGGGCTCTCATGGATATAGC	

Note: The promoter fragment of the gene was marked as “*P*”.

Supplementary Figure 1

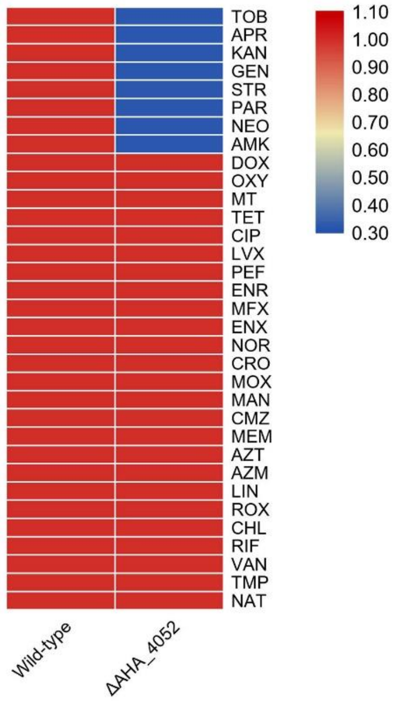

Supplementary Figure 1. Summary results of the minimum bactericidal concentration values of *A. hydrophila* wild-type and *ΔAHA_4052* strain against 33 antibiotics. TOB (tobramycin), APR (apramycin), KAN (kanamycin), GEN (gentamicin), STR (streptomycin), PAR (paromomycin), NEO (neomycin), AMK (amikacin), DOX (doxycycline), OXY (oxytetracycline hydrochloride), MT (methyloxytetracycline hydrochloride), TET (tetracycline), CIP (ciprofloxacin), LVX (levofloxacin), PEF (pefloxacin), ENR (enrofloxacin) MFX (moxifloxacin), ENX (enoxacin), NOR (norfloxacin), CRO (ceftriaxone sodium), MOX (moxalactam), MAN (cefamandole), CMZ (cefmetazole sodium), MEM (meropenem), AZT (aztran), AZM (azithromycin), LIN (lincomycin), ROX (roxithromycin), CHL (chloramphenicol), RIF (rifampicin), VAN (vancomycin), TMP (trimethoprim), and NAT (natamycin)
